# Quantifying and predicting success in show business

**DOI:** 10.1038/s41467-019-10213-0

**Published:** 2019-06-04

**Authors:** Oliver E. Williams, Lucas Lacasa, Vito Latora

**Affiliations:** 1 School of Mathematical Sciences, Queen Mary University of London, London, E1 4NS UK; 2 The Alan Turing Institute, The British Library, London, NW1 2DB UK; 3 Dipartimento di Fisica ed Astronomia, Università di Catania and INFN, I-95123 Catania, Italy; 4 Complexity Science Hub Vienna (CSHV), Josefstädterstrasse 39, A-1090 Vienna, Austria

**Keywords:** Computational science, Interdisciplinary studies

## Abstract

In certain artistic endeavours—such as acting in films and TV, where unemployment rates hover at around 90%—sustained productivity (simply making a living) is probably a better proxy for quantifying success than high impact. Drawing on a worldwide database, here we study the temporal profiles of activity of actors and actresses. We show that the dynamics of job assignment is well described by a “rich-get-richer” mechanism and we find that, while the percentage of a career spent active is unpredictable, such activity is clustered. Moreover, productivity tends to be higher towards the beginning of a career and there are signals preceding the most productive year. Accordingly, we propose a machine learning method which predicts with 85% accuracy whether this “annus mirabilis” has passed, or if better days are still to come. We analyse actors and actresses separately, also providing compelling evidence of gender bias in show business.

## Abstract (web summary)

For most actors sustained productivity defines success. Here the authors study the careers of actors and identify a "rich-get-richer" mechanism with respect to productivity, the emergence of hot streaks and the presence of gender bias, and are able to predict whether the most productive year of an actor is yet to come.

## Introduction

“It’s feast or famine in showbiz.”—Joan Rivers. A sentiment likely to be echoed by many would be stars of the silver screen. But for those that feast the rewards are, at least thought to be, worth the risk. The so-called science of success has recently uncovered many features of the careers of academics^1^, artists^2^, and all manner of other individuals whose output can be effectively assessed over the course of their working life^3–5^. For instance, in the world of scientific research it has revealed the unpredictability of the location of an academics most impactful work^1^, showing that even such prestigious awards as Nobel prizes, which usually occur later in a career^6^, are underpinned by research papers that are located randomly and uniformly throughout the ordered list of papers in the career of the awardee. On the other hand, the anatomy of funding and collaborations in universities has revealed “rich clubs” of leading institutions, and suggested that such patterns of collaborations contribute greatly to the success of these institutions, as measured in terms of over-attraction of available resources and of breadth and depth of their research products^7^. Studies of innovation in industry across different countries have found that the commercial success of manufacturing plants is far more closely related to intra-group links than external ties^8^. Strikingly, these features can be common across multiple areas; the Matthew effect^9,10^, or the rich-get-richer phenomenon, and the recently discovered presence of “hot streaks”^11^, are not restricted to isolated cases. With regards to success, a great deal of work has been done in assessing impact^1,12^, the distribution of standout or landmark works^13,14^, whether these are related to the age of the individual in question^15,16^, how impact can be assessed in the long term^17^, and even prediction of future successes^18,19^. Indeed the fortunes of both films and the actors and actresses that make them have been studied in some specific ways^17,20–22^. These studies do not however address the question that interests those who are not already on the higher rungs of the ladder of success: how can one avoid the famine and build a sustainable career in acting?

The aim of this work is to use a data-driven approach in order to define, quantify and even predict the success of actors and actresses in terms of their ability to maintain a steady flow of jobs. Drawing on the International Movie Database (IMDb), an online database of information related to films, television programs and home videos, www.imdb.com, we study the careers of millions of actors from several countries worldwide, from the birth of film in 1888 up to the present day. Each career is viewed as a profile sequence: the yearly time series of acting jobs in films or TV series over the entire working life of the actor or actress (this is similar in spirit to the approach used in^23^ to explore scientific productivity). Note that all acting jobs are considered, regardless of salary, role, screen time, or the impact of the work. The statistical analysis of such a large number of profile sequences allows us to derive some general properties of the actors activity patterns. In particular, we look at several quantities of interest such as career length, productivity (defined as the number of credit jobs in a year or in the entire career of an actor) and position of the annus mirabilis, defined as the year with the largest number of credited jobs. We also explore possible emergence of gender inequality in these properties.

The first message that emerges from our quantitative analysis is that one-hit wonders, i.e., actors whose career spans only a single year, are the norm rather than the exception. Long career lengths and high activity are found to be exponentially rare, suggesting a scarcity of resources in the acting world. These results are in agreement with previously collected evidence, pointing to the fact that unemployment rates in actors hover around 90%, and that as low as 2% of actors are able to make a living out of acting^24^. We also observe that that this dramatic scarcity unequally applies to actors and actresses, providing compelling evidence of gender bias. Moreover, the total productivity of an actor’s career is found to be power-law distributed, with most actors having very few jobs, while a few of them have more than a hundred. This indicates a rich-get-richer mechanism underpinning the dynamics of job assignments, with already scarce resources being allocated in a heterogeneous way. All of this suggests that, while activity and sustained productivity are by definition measures of performance^25^, they should in this context be considered as a proxy for success. Only a select few will ever be awarded an Oscar, or have their hands on the walk of fame, but this is not important to the majority of actors and actresses who simply want to make a living. It is the continued ability to work (as opposed to prestige) that is most likely to ensure a stable career. For these reasons we propose that predictions of success in show business should be focused on activity and productivity. Observe at this point that performance is usually conflated with success^25^. While performance is objectively measured in terms of an individual’s actions, and is typically bounded, success is traditionally measured by recognition, i.e., in terms of impact, and is a collective phenomenon which is unbounded. Notwithstanding, the severe scarcity of resources in show business forces us to redefine an actor’s success, not in terms of popularity or impact, but in terms of activity and productivity as discussed above. Incidentally, note also that being credited on IMDb is to a certain extent funnelled by recognition mechanisms such as popularity—a producer might offer the job to the actor who had the best audition or to the one who has more followers on Instagram—so productivity is not only, strictly speaking, a performance-driven indicator.

Motivated by these results, we then address the questions that interest the majority of working actors and actresses. Questions such as “am I going to get another paid job?” or “is this year going to be my best?”. We first show that efficiency, defined as the ratio between the total number of active years and the career length, is unpredictable, as there is no evident correlation between these two things. This is in line with recent studies^1^ pointing out that the most impactful pieces of work in scientific disciplines are equally likely to be located in any position throughout the entirety of an individuals output, and is therefore not predictable. Nevertheless, we here, surprisingly, find distinctive features in their temporal arrangement. In particular, we find that actor careers are clustered in periods of high activity (hot streaks)^11^ combined with periods of latency (cold streaks). Moreover, we discover that the most productive year (annus mirabilis) for both actors and actresses is located towards the beginning of their career, and that there are clear signals preceding and following the location of the annus mirabilis of an individual. Altogether, these unexpected results lead us to conclude that prediction is possible in theory. Finally, we validate this hypothesis by building a statistical learning model which predicts the location of the most productive year, finding that we can, with up to 85% accuracy, tell whether an actor’s career has reached its most productive year yet or not.

## Results

### Preliminaries

**Fig. 1:**
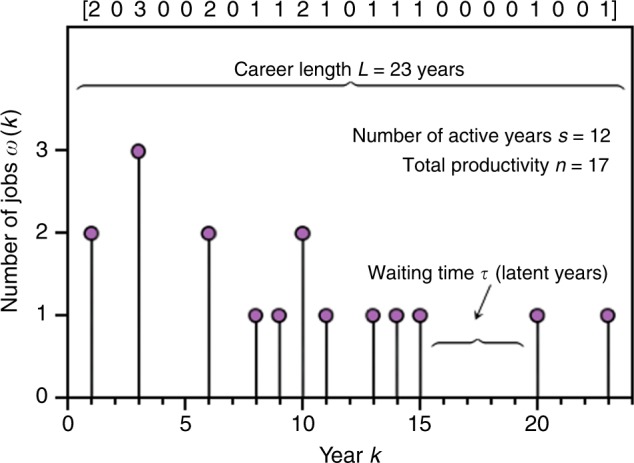
Career activity pattern of an actor. The yearly productivity of a given actor, measured as the total number of IMDb credited jobs in each year, is reported from the first to the last year of the actor activity. Shown is the case of an actor whose career spanned *L* = 23 years and who was credited a cumulated *n* = 17 different jobs in *s* = 12 years. From the yearly productivity we can construct the actor profile sequence *w*_*k*_, with *k* = 1, …, *L*, shown in brackets above the plot, which can be modelled as a stochastic marked point process

We study the careers of 1,512,472 actors and 896,029 actresses as recorded on IMDb as of January 16th, 2016, including careers stretching back to the first recorded movie in 1888. The career of each actor *a* is characterised by his/her track record, which consists of a set of pairs of numbers representing respectively each year when actor *a* was credited in IMDb, and the number of different credits in that year. As credits we count the number of acting jobs in films and/or TV series. A sketch of the typical activity pattern of an actor is reported in Fig. 1, showing the yearly credits from the first to the last year of thir career. Notice that there are not only active years, where the actor has credited jobs in IMDb, but also latent years with no recorded jobs. We therefore fill the latent years with zeros and construct the profile sequence $\{ w_k\} _{k = 1}^L$ of each actor *a* as depicted in the top part of Fig. 1. The quantity *w*_*k*_ denotes the actor’s local productivity in year *k*, i.e., the number of credited jobs in that year. The length of an actor’s career is defined as the number of years between the first and the last active year (inclusive), and is denoted as *L*. The total number of active years *s* is from now on referred to as the activity of an actor. Since a career can have latent years intertwined with active ones we must have *s* ≤ *L*, moreover *L* − *s* is the number of latent years. By definition we have: (i) *L* ≥ 1, (ii) *s* ≥ 1 and (iii) *s* = 1 ⇔ *L* = 1.

Finally, we define the total productivity *n* of an actor, as the cumulated number of credited jobs, $n = \mathop {\sum}\nolimits_{k = 1}^L {w_k}$. The annus mirabilis (AM) of a given actor is defined as the year where the actor was credited with the largest number of works in IMDb: AM = *m*, where *m* is such that $w_m = {\mathrm{max}}\{ w_k\} _{k = 1}^L$. In the case that this *m* is not unique we take the final such year: AM = max{*m*}.

### Career lengths and one-hit wonders

**Fig. 2:**
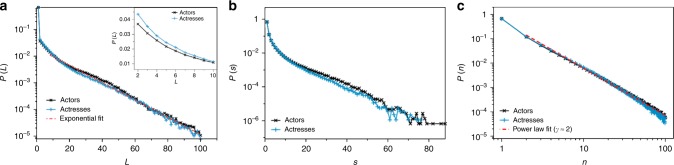
Career length, activity and productivity distributions. **a** The probability *P*(*L*) that an actor or an actress has a career of length *L*, estimated by computing the frequency histogram of the number of years between the first and the last recorded entry on IMDb. *P*(1) measures the abundance of “one-hit wonders”, namely the actors or actresses with IMDB records in a single year. A zoom for *L* ∈ [2, 10] in the inset shows that careers extending between 2 and 10 years are proportionally more frequent in women than in men. **b** Activity distribution *P*(*s*) estimated by computing the frequency histogram of the number of working years within each career (*s* ≤ *L*). Curves for actors and actresses are very similar and both exhibit a clear exponential tail, implying a "scarcity of resources". **c** Log-log plot of the total productivity distributions *P*(*n*) for actors (black) and actresses (blue). Both curves decay as a power law *P*(*n*) ~ *n*^−γ^, where *γ* ≈ 2, revealing a Zipf’s law for the total number of acting jobs

We start our analysis by exploring the statistics of the career length *L*. In Fig. 2a we plot in a semi-log scale the empirical distribution of career lengths *P*(*L*), for both actors and actresses finding that the tail is well fitted by an exponential distribution. By construction, *P*(*L* = 1) = *P*(*s* = 1) and this quantity represent the percentage of one-hit wonders i.e., of actors whose career started and ended, according to IMDb, in the same year. Interestingly, we find that the percentage of such cases is extremely high (around 69% for males and 68% for females) and deviates from the otherwise decaying exponential distribution. This sharp deviation highlights that one-hit wonders are not an exception in show business, but, on the contrary, are the norm^26^. A zoom of the distribution in the range *L* ∈ [2, 10] is reported in the inset of (a), revealing systematic differences between actors and actresses, suggesting that it is consistently more common to find (non-one-hit wonder) actresses with shorter career lengths than actors. We have indeed performed a model selection experiment which confirms that gender bias is statistically significant (see Supplementary Note 1 for details).

The empirical probability distribution of activities, displaying the probability of sampling an actor that worked in *s* years, is shown in Fig. 2b in a semi-log scale. Most of the actors and actresses are only active in a single year (*s* = 1), as by default $s = 1 \mapsto L = 1$. The probability of finding actors with large activity, i.e., those that have worked in many different years, decays exponentially fast. This exponential decay mimics the similar decay in the probability of finding long career lengths and altogether are the basis for claiming a scarcity of resources in show business, i.e., there are many more actors/actresses than job offers^27^. This lack of resources naturally leads to a question: how are they allocated? We address this question in the next section.

### Productivity and the rich-get-richer phenomenon

Figure 2c shows the empirical distributions of total productivity *P*(*n*), reporting the normalised numbers of actors or actresses with *n* appearances in movies or TV series over their careers. While the career length distribution *P*(*L*) and the activity distributions *P*(*s*) are well fitted in their tails by an exponential law, the function *P*(*n*) decays more slowly and can be fitted by a power law *P*(*n*) ~ *n*^−*γ*^ with exponent *γ* ≈ 2. Notice that similar behaviours have already been found in the context of two-mode actor-movie networks and of other systems that can be modelled as bipartite graphs^28^. A power law in the distribution of total productivity implies also the existence of scaling in the rank-frequency distribution of productivity. It is indeed well known that observing a power-law distribution with exponent *γ* for the abundance of some variable is equivalent to obtaining a power-law scaling for the frequency of the variable that appears with rank *r*: *f*(*r*) ~ *r*^−*α*^^29^. The exponents of the two scaling laws are mathematically related via *α* = 1/(*γ* − 1). The celebrated Zipf’s law refers to the particular case of an exponent *α* ≈ 1, which is indeed the case here. In turn, the emergence of a Zipf’s law for the rank-frequency distribution of the total productivity of an actor suggests a possible mechanistic explanation for our observations. Many different proposals for the mechanism underpinning the emergence of a Zipf’s law, and several names for the phenomenon itself, have been put forward in various contexts, including the Simon–Yule process, the mechanism of preferential attachment, the Matthew effect, the Gibrat principle, rich get richer, etc.

In this context, we can suggest a possible mechanism for the onset of a power-law distribution for the total productivity in terms of a rich-get-richer phenomenon. Let us consider a generative model of a bipartite graph whose two sets of nodes represent respectively actors and movies. Actors acquire new links to movies, thus increasing their productivity, if they get a role in those movies. Suppose all actor nodes start with zero edges and acquire their first edges only according to a fitness, that is initially assigned at random or on some hypothetical intrinsic acting skill. When more movie nodes enter the network, actor nodes that acquire new edges gain popularity and this, in turn, increases their fitness. As it is well known that producers are more keen to offer a role to popular actors, actor nodes with high fitness are more likely to attract new edges. This leads to a multiplicative effect which clearly expresses the rich-get-richer phenomenon; actors with many job assignments will have a higher chance of working even more than actors with low productivity. In conclusion, the same rich-get-richer mechanism, which is at the heart of networks with power-law degree distributions^30–33^, can also be the cause of the observed power laws in the total productivity of movie actors. This result is not at all unexpected, after all, the more well-known an actor is, the more likely producers will want him or her in their next film, if only for commercial purposes. What is perhaps dramatic about this observation is that it is well known that rich-get-richer effects are rather arbitrary and unpredictable, as large hubs can evolve out of unpredictable and random initial fluctuations which have been amplified, and not based on any particular intrinsic fitness^33^ (such as acting skills). Quoting Easly and Kleinberg: “if we could roll time back 15 years, and then run history forward again, would the Harry Potter books again sell hundreds of millions of copies, or would they languish in obscurity while some other works of children’s fiction achieved major success?”. As a matter of fact, it seems likely that across different parallel universes productivity would still have a power-law distribution, but it is far from clear that the most productive actors would always be the same. Interestingly, this hypothesis has recently been validated in an online social experiment for the case of musical popularity^34^. In summary, productivity is probably the variable every actor aims to maximise, but these results suggest that boosting productivity can be more of a network effect^35,36^ than a consequence of acting skills.

### Efficiency is unpredictable

In Fig. 2 we observed that career length *L* and activity *s* are variables which are both exponentially distributed, indicating a scarcity of resources. In this section we further explore whether the two quantities *L* and *s* are correlated. We first define an actor’s efficiency as the ratio *s*/*L* of active years over the entire career, and we investigate how the efficiency is distributed. The results reported in Supplementary Fig. 1, show that: (i) the efficiency distribution drops rapidly as *s*/*L* approaches either zero or one –i.e., most actors and actresses have intermediate values of efficiency– and that (2) for middle-range efficiency the distribution is essentially uniform (see Supplementary Note 2 for additional details). This suggests that efficiency is not predictable and that, for middle-range efficiency, the only correlations that emerge between the activity *s* and the career length *L* come from the fact that, by construction, *s* ≤ *L*. To further validate this, we performed a scatter plot of *s* versus *L* for all actors and actresses, and computed the Pearson correlation coefficient, then compared this to the correlation coefficient of a null model generated by randomly extracting values of *L* and *s* from the pool of career profiles, ensuring that *L* ≥ *s* (Supplementary Fig. 2). For actors, *s* and *L* exhibit a Pearson correlation coefficient *r* ≈ 0.69, whereas in the null model we obtained *r*_null_ ≈ 0.6. In the case of actresses we found *r* ≈ 0.69 and *r*_null_ ≈ 0.58. As expected, *s* and *L* are indeed correlated quantities, but the correlations can almost entirely be explained by a null model. In other words, for intermediate ranges there are no additional correlations between length and activity: the activity of actors cannot therefore be predicted by their career length, and we can conclude that the efficiency is an unpredictable quantity.

### Actors careers are clustered in hot and cold streaks

**Fig. 3:**
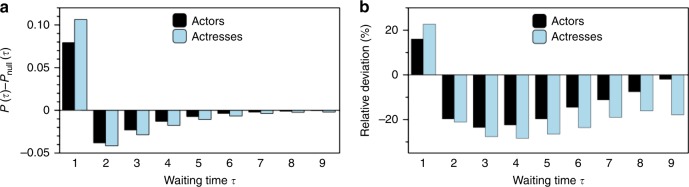
Waiting time distribution. **a** Difference *P*(*τ*) − *P*_null_(*τ*) between the waiting time distribution in the profile sequences and in a randomised null model, for actors (black bars) and actresses (blue bars). Systematically short waiting times, *τ* = 1, are overrepresented with respect to the null model, while the opposite is true for intermediate waiting times *τ* > 1. **b** The percentage relative difference [*P*(*τ*) − *P*_null_(*τ*)] ⋅ 100/*P*_null_(*τ*) reveals a notable difference between actors and actresses: cold streaks fade away faster for actors

To understand the temporal arrangement of active years within the profile sequence of a given actor, we now consider the statistics of waiting times. A waiting time *τ* is defined as the time elapsed (in years) between two active years (equivalently, a waiting time is a collection of successive latent years), and its statistics provide a classical way to analyse the presence of memory and bursts in time series^37,38^. We have estimated the waiting time distribution *P*(*τ*) for actors and actresses, discarding those with short career lengths, *L* < 10 years, to avoid a lack of statistics. To estimate this distribution, for each actor (actress) we count how frequently one observes waiting times of a certain duration *τ*, and normalise the accumulated frequencies. This process will inevitably introduce finite size biases since, for short career lengths, we are more likely to find short waiting times, simply because there is no room for long ones. For a proper comparison we therefore have also computed the distribution for a randomised null model *P*_null_(*τ*) where all of the profile sequences have been shuffled (while keeping the first event *w*_1_ and the last event *w*_*L*_ unaltered). A lack of temporal correlations would imply *P*_null_(*τ*) = *P*(*τ*), whereas systematic differences suggest the onset of temporal correlations in the activity of actors. In panel (a) of Fig. 3 we report the difference *P*(*τ*) − *P*_null_(*τ*) as a function of *τ*.

For both actors and actresses, we systematically find *P*_null_(*τ* = 1) < *P*(*τ* = 1), and *P*_null_(*τ* > 1) > P(τ > 1), that is, active years are more clustered than they would be by chance, and hence the same is true for periods of inactivity. This means that the profile sequence shows *clustering* and is composed of bursts of activity (hot streaks) where actors and actresses are more likely, than would be expected by chance, to work in a year if they worked the year before (*τ* = 1). This result is in agreement with recent findings in other creative jobs in science and art^11^. Additionally, these hot streaks are interspersed by abnormally long periods of latency (cold streaks) where authors are less likely than random to work in a given year if they did not work the year before (*τ* > 1).

Furthermore, to appropriately compare deviations from the null model for different waiting times, in Fig. 3b we plot the relative difference (in percentage) [*P*(*τ*) − *P*_null_(*τ*)] · 100/*P*_null_(*τ*). We find a substantial difference between actors and actresses: while deviation from the null model decays for larger waiting times *τ* in the case of actors, for actresses this relative deviation is maintained, pointing to a longer memory kernel, in turn suggesting that having a period of latency is overall more detrimental for actresses than for actors.

### Predicting the annus mirabilis

It has recently been found that the most impactful publication that a scientist will produce is equally likely to occur at any stage of their career^1^. Here we explore a related question in the context of actors and actresses. Instead of impact, the indicator of success under study is productivity, as measured by the number of credited works in IMDb. We concentrate on actors and actresses with working lives extending beyond *L* = 20 years. We restrict our reported results to those cases where there were at least 5 credited jobs in the annus mirabilis (AM), although other thresholds do produce qualitatively similar results. The subset of actors with *L* > 20 and more than 5 acting jobs in the AM consists of 15357 actors (1.02%) and 5904 actresses (0.65%). The large gender difference indicates that actors tend to have more acting jobs than actresses.

**Fig. 4:**
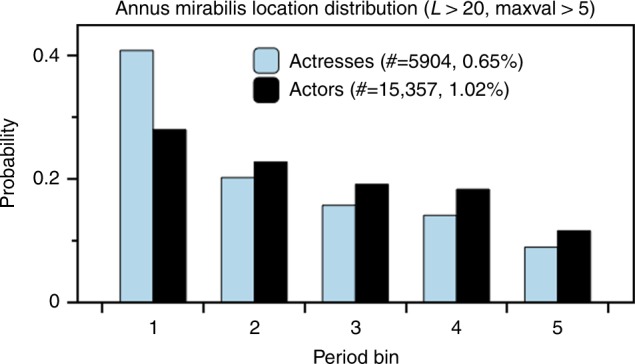
Annus mirabilis tends to occur sooner rather than later. Position of AM within an actor or actress’s career, where the career length is binned into 5 bins in every case, to be able to compare profiles of different career lengths. We systematically find that the most probable location of the annus mirabilis is towards the beginning of a career, although this effect is considerably more acute in the case of actresses

In Fig. 4 we plot the probability with which the AM will occur at each point within an actor or actress’s career. To be able to compare these probabilities over careers of varying lengths, we have broken up each actor’s time series of *L* years respectively into 5 bins (other segmentations produce qualitatively similar results). The plots consistently indicate that the most probable location of the annus mirabilis is towards the beginning of a career. Although the results are qualitatively similar for male and female actors, this bias is much more pronounced in the case of actresses, further confirming the gender difference previously observed.

**Fig. 5:**
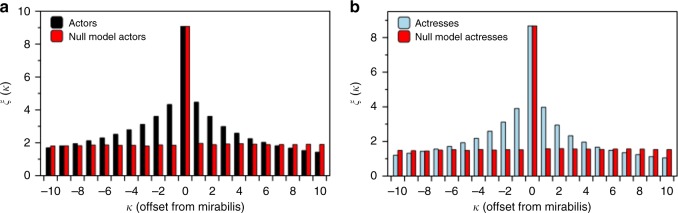
The annus mirabilis is predictable. The total number of acting jobs, *ξ*(*κ*), averaged over all **a** actors and **b** actresses, is reported as a function of the number of years *κ* after or before the annus mirabilis. Only actors and actresses with a career lasting more than *L* = 20 years and annus mirabilis with *w* > 5 acting jobs have been selected. In both cases, we observe a clear non-monotonic pattern, indicating that the annus mirabilis is either approaching or has just passed. For comparison, we report in red the results obtained for a null model where the profile sequences of all actors and actresses have been shuffled. No pattern emerges in that case

To study whether one can detect the imminent appearance of an actor’s annus mirabilis we have analysed, for both actors and actresses, the average number of acting jobs before and after the AM. In order to do this consistently, we initially perform a translation $k \, \mapsto \, \kappa$ that aligns all profile sequences, so that the annus mirabilis *k* = y^*^ all occur at *κ* = 0. We then define:

$$\xi (\kappa ) = \frac{1}{{|A|}}\mathop {\sum}\limits_{i = 1}^{|A|} {w_{y^ \ast + \kappa }^{(i)}} ,$$

where *κ* is the offset from the annus mirabilis and |*A*| is the size of the set of actors/actresses for which there exists a profile sequence with an input at offset *κ*. In Fig. 5 we plot *ξ*(*κ*), showing that, on average, there is a clear increase in the number of jobs preceding the AM and a clear decrease immediately afterward. This pattern is absent in the corresponding null models obtained by shuffling the profile sequences (red bars).

It is interesting to note that similar patterns have been observed before in the context of scientific productivity, although recent research challenges this paradigm^23^. As a matter of fact, in^23^ the authors leveraged the observed shapes of scientific productivity profiles and followed an unsupervised learning approach to cluster different types of careers. Here, instead, we shall follow a supervised learning approach and will now show how the observed patterns can indeed be exploited to build a method for the early prediction of the annus mirabilis.

Based on our observed distribution of jobs surrounding the annus mirabilis we initially propose a naive early-warning criterion: if the career sequence is non-monotonic around a value of *k*, i.e., if *w*_*k*_ > *w*_*k*−1_ and *w*_*k*+1_ < *w*_*k*_, then the year *k* is a good candidate for the annus mirabilis. With this criterion in mind, one could ask the following question: given a sample of an actor or actress’s profile sequence, can we tell whether the annus mirabilis has already passed or not? Mathematically, the question above can be formalised as follows: given a career sequence $(w_k)_{k = 1}^L$ such that the maximal total productivity occurs at time *k* = y^*^, consider a truncated sequence $\bar w_k = (w_k)_{k = 1}^{T \le L}$. We now wish to know if we can accurately assess whether y^*^ ∈ {1,...,*T*} using only $\bar w_k$. This forms a binary classification problem, in which $\bar w_k \in {\cal{C}}_1$ if $y^ \ast \, \notin \, \{ 1,...,T\}$ and $\bar w_k \in {\cal{C}}_2$ otherwise. Our naive criterion, as illustrated above, readily provides the heuristic: $\bar w_k \in {\cal{C}}_1$ if $\bar w_k$ is monotonic, and $\bar w_k \in {\cal{C}}_2$ if not. When this method is tested on an appropriately generated set ${\cal{W}}$ of truncated sequences (see Supplementary Note 3 for details) we find that it is correct ~69.2% of the times for actors, and ~75.0% of the times for actresses. This model now forms a benchmark against which we will test a more refined approach. The idea is to relax our classification method by introducing some parameters which allow for deviation from the rigid heuristic, then train those parameters on some subset ${\cal{T}}\subsetneq {\cal{W}}$, and subsequently test the trained model on the test set ${\cal{W}}\backslash {\cal{T}}$. To do this let us first define the function

1 $$D\left( {\bar w_k} \right) = - \mathop {\sum}\limits_{y = 1}^{T - 1} {{\mathrm{min}}} \left( {0,\bar w_{y + 1} - \bar w_y} \right).$$

At each year *k* the contribution to *D* from that year is zero if the total productivity in the subsequent year is larger. This means that for a monotonically increasing sequence $\bar w_k$, $D\left( {\bar w_k} \right) = 0$. If productivity decreases from year *k* to *k* + 1, then *D* will increase by a corresponding amount.

$D\left( {\bar w_k} \right)$ effectively measures how far the sequence $\bar w_k$ is from being monotonically increasing, thus we can use it to relax our naive heuristic by defining some threshold *d* such that the decision rule $C\left( {\bar w_k,d} \right)$ becomes

2 $$C\left( {\bar w_k,d} \right) = \left\{ {\begin{array}{*{20}{c}} {{\cal{C}}_1} & {{\mathrm{if}}\,D\left( {\bar w_k} \right) \, < \, d} \\ {{\cal{C}}_2} & {{\mathrm{if}}\,D\left( {\bar w_k} \right) \ge d.} \end{array}} \right.$$

This new classifier is more flexible than the naive heuristic as we have introduced a parameter *d* which can now be optimised (trained) as follows: if we denote $C^ \ast \left( {\bar w_k} \right)$ as the true class of the sequence $\bar w_k$, then the optimal value of the parameter d^*^ is the value of *d* that minimises the following loss function

3 $${\cal{L}}\left( {{\cal{T}},d} \right) = - \mathop {\sum}\limits_{\cal{T}} \delta \left( {C\left( {\bar w_k,d} \right),C^ \ast \left( {\bar w_k} \right)} \right).$$

Where *δ*(*X*, *Y*) yields one if *X* = *Y* and 0 otherwise. This value for d^*^ is then used to classify the remaining sequences in ${\cal{W}}\backslash {\cal{T}}$. The results of this testing on both actors and actresses can be partially summarised by the two confusion matrices CO_*m*_ (for actors) and CO_*f*_ (for actresses):

4 $${\mathrm{CO}}_m = \left[ {\begin{array}{*{20}{c}} {33775} & {5659} \\ {10771} & {52000} \end{array}} \right],\,{\mathrm{CO}}_f = \left[ {\begin{array}{*{20}{c}} {12549} & {2593} \\ {3596} & {26682} \end{array}} \right]$$

The classical metrics used to assess the performance of the classifier, namely accuracy, precision, recall and the F1 score, are summarised in Table 1. We find that the accuracies of the prediction are 84 and 86% respectively, i.e., ~10% higher than those obtained using a naive heuristic.

**Table 1:** Performance metrics (accuracy, precision, recall and F1 score) of the proposed classification method for the prediction of the annus mirabilis

Quantity	Actors	Actresses
Total ${\cal{C}}_1$	44652	16145
Total ${\cal{C}}_2$	57553	29275
Accuracy	0.8405	0.8637
Precision	0.8608	0.8287
Recall	0.7575	0.7773
F1 score	0.8058	0.8021

**Fig. 6:**
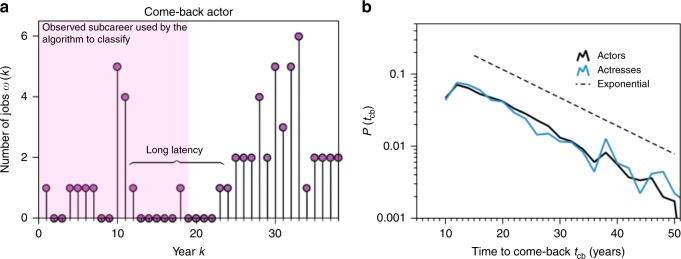
Comebacks of actors are unpredictable. **a** Typical profile sequence of an actor exhibiting a comeback after a long period of latency. Such cases might lead to misclassification when the subcareer fed to the prediction algorithm (highlighted in pink) captures a long latency period: the prediction algorithm wrongly classifies the pink sequence as one where the annus mirabilis has passed. **b** Semi-log probability distribution of the estimated time lapse from the (wrongly estimated) annus mirabilis to the true one (i.e., the time *t*_cb_ to come-back for actors with profile sequences such as the one in panel a), for those misclassified samples where the algorithm wrongly predicts that the annus mirabilis had already passed (a linear binning has been applied to the data). Modelling the position of the secondary peak (comeback burst) as a random variable, the fact that *P*(*t*_cb_) decays exponentially, suggests that this random variable is memoryless (Poisson process), i.e., the comeback burst is intrinsically unpredictable

To round off, we have further explored the nature of the ≈15% of samples which are misclassified (see Supplementary Note 3 for details). We found that false negatives (samples for which the annus mirabilis is wrongly predicted to be still yet to come) arise due to the conservative nature of the prediction model, hence more refined versions of the prediction model might yield even better prediction results (Supplementary Fig. 3). Conversely, we find that false positives –where the annus mirabilis is wrongly predicted to have passed– are usually related to actors and actresses experiencing a come-back at a later stage of their careers (see Fig. 6a for an example). Interestingly, the positions of these late bursts of activity seem to be fundamentally difficult to predict (Fig. 6b).

## Discussion

In this work we have made use of the vast quantity of data presented by IMDb to explore, analyse and predict success on the silver screen. By studying the careers of 1,512,472 actors and 896,029 actresses from 1888 up to 2016, we have uncovered a number of distinctive patterns which include an endemic scarcity of resources, a rich-get-richer mechanism of job assignation, the onset of hot and cold streaks of productivity^11^ and an annus mirabilis which can indeed be predicted. Such patterns – which we show to systematically differ for actors and actresses, suggesting strong evidence of gender bias^26 ^– not only allow us to identify qualities of individual actors or actresses working lives, but also to gain a deeper insight into the mechanisms by which jobs are themselves assigned, where high productivity is not necessarily based on merit and is likely to be a network effect^34–36^. Based on our findings, we have then constructed a statistical learning model that predicts with up to 85% accuracy whether an actor or actress is likely to have a brighter future, or if the best days are, unfortunately, behind them. While we expect refined versions of the prediction model to give even higher accuracy, it is worth noting that actors with long latency periods who then experience late comebacks are rare but intrinsically difficult to predict.

We hope that the methods presented and the results obtained will contribute to the new science of success^35^. Given the scope of our findings across the industry, we also wish that our article will be of interest to those working in show business.

## Supplementary information

Supplementary Information

## Data Availability

Data is available upon request, or can be accessed at 10.17605/OSF.IO/NDTA3

## Code Availability

Codes are available upon request.
