# Supplementary material for: Quantifying and predicting success in show business
Source: Nat Commun. 2019 Jun 4;10:2256. doi: 10.1038/s41467-019-10213-0 (PMC6548779; doi:10.1038/s41467-019-10213-0)
Supplement: Supplementary file 1 — Supplementary Information [file 41467_2019_10213_MOESM1_ESM.pdf]

Supplementary information: Quantifying and predicting success in show business

Williams et al.

Supplementary note 1: A model selection scheme to confirm gender bias

Gender bias is confirmed by observing quantifiable differences across the different measures observed in this work. Whereas for some of these measures differences are obvious (e.g. for the case of waiting time statistics or the location of the annus mirabilis, see Figs 4 and 5 in the main part of the manuscript), for other measures, such as career lengths (Fig 1) these differences are more subtle, at least to the naked eye. To further confirm that the observed differences in career length distribution $P(L)$ are indeed statistically significant, we have performed the following statistical experiment: (i) first, we fit $P(L)$ for male and female to respective exponential distributions for $L \in [2, 100]$ (discarding $L = 1$ in the fit, which is a clear outlier), obtaining two exponential functions $g_m(x) = c_m \exp(\alpha_m x)$ and $g_f(x) = c_f \exp(\alpha_f x)$ for actors and actresses respectively. (ii) Then, treating the estimated $P(L)$ as if it was an empirical dataset, we merge together the datasets for men and women ($N = 198$ points), and then fit this merged dataset to a new exponential function $g_h(x) = c_h \exp(\alpha_h x)$. The rationale is that if the observed differences between actors and actresses were spurious, then the fit for the merged dataset would be a superior model to the separate fits for actors and actresses, from a model selection scheme. To assess this, we then compare two statistical models which aim to fit the merged dataset: the first model (M1) is simply $g_h(x)$ and fits the merged dataset, whereas the second model (M2) is

a *combination* of $g_m(x)$ (which applies to the part of the merged dataset which refers to actors) and $g_f(x)$ (which fits the subset of the data pertaining to actresses). Model selection is finally based on minimizing the Akaike Information Criterion (AIC) (*1*).

We obtain the following results: fits are

$$g_m(x) = 0.02382\exp(-0.07578x), g_f(x) = 0.02232\exp(-0.07768x),$$

$$g_h(x) = 0.02306\exp(-0.07673x),$$

and the AIC of each statistical model is AIC$(M_1) = -621.63$, AIC$(M_2) = -654.65$. Since AIC$(M_2) <$ AIC(M_1) (relative likelihood of M_2 with respect to M_1 is $\exp([$AIC$(M_1) -$ AIC$(M_2)]/2) \approx 1.5 \cdot 10^7$), we conclude that the gender differences are genuine. Similar results are found for other metrics (data not shown).

Supplementary note 2: Efficiency: additional details

By construction, we have $s \leq L$, hence the number of active years is at most equal to the career length, and if inactive years are present between active years, then we will have $s < L$. Also, $L = 1$ implies $s = 1$, and thus in this case we have $s/L = 1$. As roughly 68% of actors are one-hit-wonders, we expect that the probability that an actor has optimal efficiency $P(s/L = 1) \approx P(L = 1)$. However, it is clear that these are 'pathological' cases as efficiency is not really well defined for one-hit wonders. In what follows, we therefore assume that the case ($s = 1$, $L = 1$) is an outlier with regards to the analysis of efficiency.

$P(s/L)$

In Supplementary Figure 1 we plot $P(s/L)$ on semi-log axes for actors (top panel) and actresses (bottom panel). As might be expected, the distribution decreases rapidly as s/L approaches either zero or one, suggesting that most actors and actresses have intermediate values of efficiency (see SI for a discussion and a heuristic explanation of this phenomenon). The shape of $P(s/L)$ in the intermediate range is fractal-like, which is due to the fact that s and L are (small) integers and thus s/L cannot take arbitrary values in $[0, 1]$. The fractal shape can actually be related to the density of irreducible fractions over the integers, as depicted in the bottom panel of Figure 1, and is not a property linked to the relation between s and L. In other words, when this effect is factored out, then $P(s/L)$ is essentially flat in the intermediate range.

$P(s/L)$ is small for $s/L \to 0$ and $s/L \to 1$: a discussion

Here we provide an heuristic explanation for the fact that $P(s/L)$ quickly drops to zero when for $s/L \to 0$ and $s/L \to 1$:

(1) Let us assume first that L is large. In that case s should not be much smaller than L simply because that would imply an actor has been indefinitely in the business without barely working,

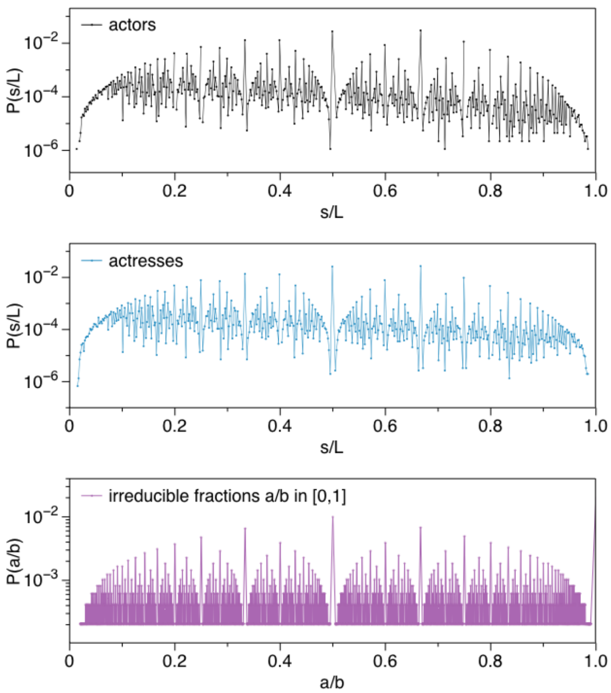

Supplementary Figure 1: **Distribution of actor efficiency**. The probabilities of finding actors (top panel) and actresses (middle panel) with a given value of the ratio s/L are shown as a function of s/L. The curves have been computed after homogeneously binning the support [0,1] into 500 bins. The fractal shape of the curve is reminiscent of the binned density of irreducible fractions $a/b,\ b > a$, shown in the bottom panel. No obvious differences emerge here between actors and actresses.

which is probably not economically viable. On the other hand s should not be of the same order as L (i.e. $s/L \approx 1$) because that would imply that the actor has an abnormally large activity, contradicting the scarcity of resources hypothesis evidenced by Figure (2) in the main text. Thus when L is large, s/L is likely to take values only in an intermediate range.

(2) Let us now assume that L is small (but larger than one). In that case s should not be too close to L since, if that were the case, this would mean that an actor with a very promising career has quit, something that a priori is unlikely. On the other hand, s/L should not be too low since $s \geq 1$, so a trivial bound is $s/L \geq 1/L$, which is a large lower bound if L is small. Hence the only likely outcome when L is small is that, again, s/L should be in an intermediate range.

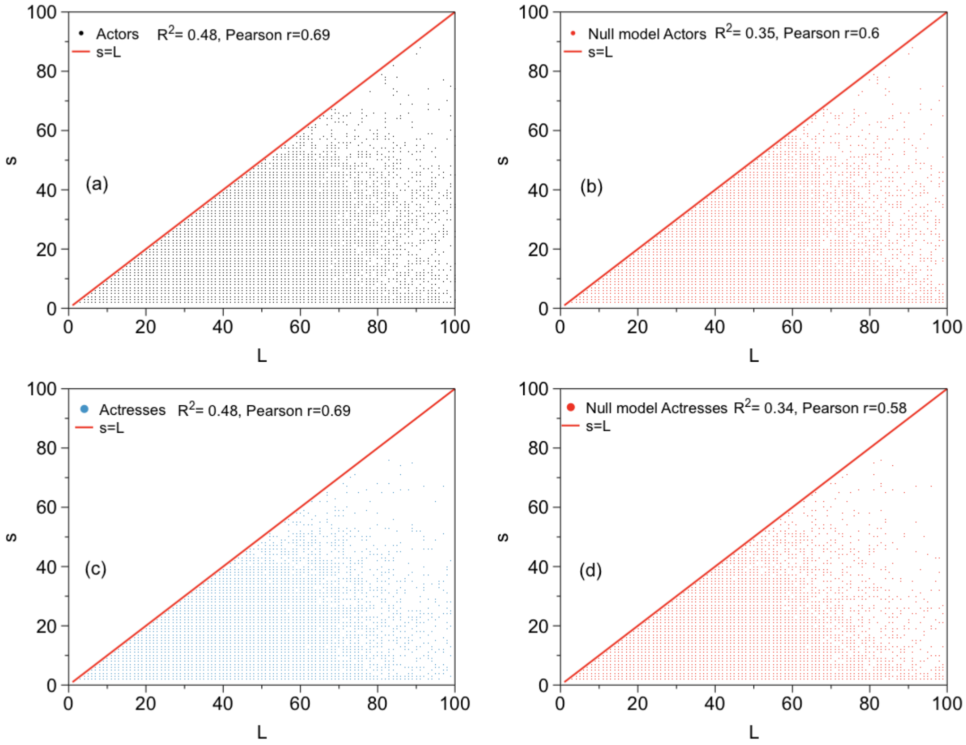

Supplementary Figure 2: **Scatterplots of activity vs career length.** The activity value s is reported versus the the career length L for each actor and actress (panels (a) and (c)). Results are compared to those (panels (b) and (d)) obtained with a null model with no correlations other than those due to the constraint $s \leq L$.

Scatter plots

To further confirm that s and L exhibit weak correlations, and therefore to show that the efficiency of an individual is essentially unpredictable, we have constructed the scatter plots of s vs L and we have computed the Pearson correlation coefficient r between the two variables. Panels (a) and (c) in Figure 2 report the results obtained respectively for actors and actresses. By construction, $s \leq L$. If no further correlations between activity and career length are present, then the scatter plot should cover, to some extent, the lower triangular area, and r would be necessarily positive accordingly.

In order to understand whether the observed patterns are due solely to the constraint $s \leq L$, we have generated a null model by randomly rewiring the empirical pairs (s, L) while forcing the shuffled pairs that still respect the condition $s \leq L$. In other words, we Monte Carlo sample pairs (s, L) and (s', L'), and with a certain probability we swap and construct (s', L) and/or (s, L'), provided that within each new pair the rule $s \leq L$ holds. In this way, the marginal distributions $P(s)$ and $P(L)$ are conserved, while the couples s and L are uncorrelated by construction. We notice that the scatterplots obtained with the null model and reported in panels (b) and (d) exhibit the same patterns found in panels (a) and (c). This result is strengthened by the Pearson correlation coefficient, which is equal to 0.69 for both actors and actresses, a value only slightly larger than the values of 0.60 and 0.58 found in the two corresponding null models. Hence, we can conclude that most of the correlations between s and L can be explained by a null model, and therefore the two quantities s and L are uncorrelated, i.e. the efficiency s/L is unpredictable.

Supplementary note 3: Prediction model: additional details

Generating truncated career sequences

Given a set $\mathcal{A}$ of either actors or actresses, we wish to build a set $\mathcal{W}$ of "subcareers" modelling the career-to-date of each actor/actress up to some point, this being a necessary setup to build an *early prediction* model. Accordingly, for both training and testing tasks, we need to construct truncated career sequences with an assigned label describing whether the location of the annus mirabilis (AM) happens inside the truncated sequence or at a later stage. To do this, let us first assume that $\mathcal{A}$ has had any conditions on careers already applied (e.g. no sequences of length less than 20, no sequences with AM less than 5). Then for each actor $a \in \mathcal{A}$ we generate 5 random variables T_i ($i = 1 \dots 5$) uniformly sampled from the interval $[1, L_a]$, where L_a is the career length of of actor a, and take note of whether each T_i occurs before or after the AM for that actor/actress. For each actor a, one then constructs 5 different subcareer series $\omega_i^a = \{(w_{1,i}^a, w_{2,i}^a, \dots, w_{T_i,i}^a)\}$, and then $\mathcal{W}$ is just the union of all these sequences

$$\mathcal{W} = \bigcup_{a,i} \omega_i^a$$

This gives us a set of $5 \cdot |\mathcal{A}|$ subcareers with known pre/post AM classification.

Misclassification analysis

The prediction model correctly classifies around $84 - 85\%$ of the samples. Since classes are balanced, this prediction accuracy is a meaningful indicator of the performance of the classification. Now, what happens with the $15 - 16\%$ of misclassified samples? Can we gain any understanding as to why the model misclassifies those cases?

A first observation is that, by construction, the algorithm 'observes' truncated sequences and uses these to make its decision. Now, the size of these observation windows, i.e. the size of the initial portion of the career that the algorithm uses to make the prediction as to whether

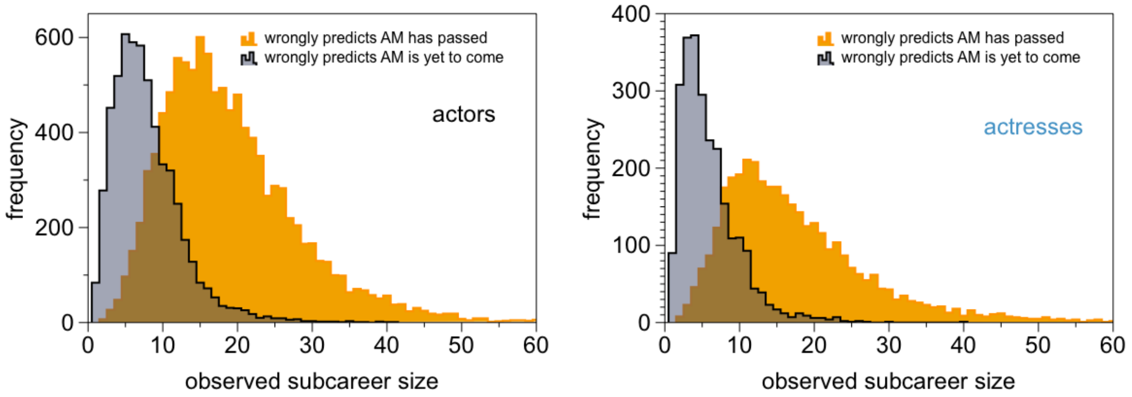

Supplementary Figure 3: **Short observation windows affect misclassification.** Frequency histograms of the truncated subcareer sizes the algorithm is given to make a decision. We see that observed window sizes are typically smaller for 'false negatives' than for the rest of the misclassified samples. Left panel is for actors, while right panel refers to actresses.

the annus mirabilis (AM) has passed or not, is by construction a random variable itself. Accordingly, in some cases this size can be rather small, and one could expect that the prediction model does not have enough information to make a sound classification. We conjecture that this effect is at the heart of many of the misclassified samples associated to 'false negatives', i.e. misclassified samples where the algorithm wrongly predicts that the annus mirabilis is yet to come. To assess this, we have explored, for each misclassified sample, the actual size of the observed window, and plotted the frequency histogram in Figure 3. Interestingly, we find that the observed window size is *systematically shorter* for the samples for which the algorithm wrongly predicts that the annus mirabilis is yet to come. This confirms our hypothesis: in other words, this misclassification is not related to difficulties of the prediction imposed by the data, but is due to the setup of the prediction model.

On the other hand, we have confirmed that for most of the 'false positives', i.e. samples where the algorithm wrongly predicts that the AM has passed, this happens because actors make an unexpected comeback later in their career. These cases are therefore intrinsically difficult to

predict. A typical example of a false positive is reported in the left panel of Figure 6. For this specific case the algorithm is given a subcareer consisting on the first 16 years of the actor's career. Because a notable burst has passed the algorithm wrongly predicts that the annus mirabilis has passed. However, after a long latency period, the career of the actor exhibits a comeback with a secondary burst of activity including the true annus mirabilis.

To further understand this class of behaviours, we might wonder whether the occurrence of these secondary bursts or comebacks is itself predictable. To explore this, for each misclassified false positive sample we measure the come back time t_{cb}, computed as the time distance between the wrongly estimated annus mirabilis and the true one. If the onset of comeback bursts was unpredictable, we could model it as a memoryless Poisson process, and for that case one should expect that the waiting-time distribution $P(t_{cb})$ should be exponentially decaying. We have indeed confirmed that this is the case for both actors and actresses (see right panel in Figure 6). This result further supports the hypothesis that comebacks are intrinsically difficult to predict.

Supplementary Reference

1. K.P. Burnham, D.R. Anderson, *Model Selection and Multimodel Inference: A practical information-theoretic approach* (2nd ed.) (Springer-Verlag, 2002).
